# The olfactory limbus of the red fox (*Vulpes vulpes*). New insights regarding a noncanonical olfactory bulb pathway

**DOI:** 10.3389/fnana.2022.1097467

**Published:** 2023-01-10

**Authors:** Irene Ortiz-Leal, Mateo V. Torres, Víctor Vargas-Barroso, Luis Eusebio Fidalgo, Ana María López-Beceiro, Jorge A. Larriva-Sahd, Pablo Sánchez-Quinteiro

**Affiliations:** ^1^Faculty of Veterinary, University of Santiago de Compostela, Lugo, Spain; ^2^Cellular Neuroscience, IST Austria (Institute of Science and Technology Austria), Klosterneuburg, Austria; ^3^Institute of Neurobiology, Universidad Nacional Autónoma de México, Querétaro, Mexico

**Keywords:** olfaction, fox, Canidae, immunohistochemistry, lectins, olfactory limbus

## Abstract

**Introduction:** The olfactory system in most mammals is divided into several subsystems based on the anatomical locations of the neuroreceptor cells involved and the receptor families that are expressed. In addition to the main olfactory system and the vomeronasal system, a range of olfactory subsystems converge onto the transition zone located between the main olfactory bulb (MOB) and the accessory olfactory bulb (AOB), which has been termed the olfactory limbus (OL). The OL contains specialized glomeruli that receive noncanonical sensory afferences and which interact with the MOB and AOB. Little is known regarding the olfactory subsystems of mammals other than laboratory rodents.

**Methods:** We have focused on characterizing the OL in the red fox by performing general and specific histological stainings on serial sections, using both single and double immunohistochemical and lectin-histochemical labeling techniques.

**Results:** As a result, we have been able to determine that the OL of the red fox (*Vulpes vulpes*) displays an uncommonly high degree of development and complexity.

**Discussion:** This makes this species a novel mammalian model, the study of which could improve our understanding of the noncanonical pathways involved in the processing of chemosensory cues.

## Introduction

Modern neuroanatomical, electrophysiological, and genomic studies have revealed that the concept of a single olfactory system is an oversimplification. Instead, the seemingly simple mammalian nasal cavity contains a significant number of olfactory systems, some of which have only been discovered relatively recently (Barrios et al., 2014a). The mammalian olfactory system can be divided into several subsystems based on the anatomical locations of the involved neuroreceptor cells, the receptors families that are expressed, the signaling transduction mechanisms employed, the chemosensory stimuli detected, and the targets of the sensory neuron axons within the rhinencephalon (Munger, 2009). However, growing evidence supports synergistic and cooperative interactions among these olfactory subsystems (Mucignat-Caretta et al., 2012; Pardo-Bellver et al., 2022).

The best-studied systems are the main olfactory system (MOS) and the vomeronasal system (VNS), which are both found in most mammalian groups, with a few exceptions (Trotier and Døving, 1998). The olfactory receptors (ORs) of the MOS are located in cilia within the mucosa lining the ethmoidal turbinates and in the caudal portion of the nasal septum (Salazar et al., 2019). Neuroepithelial axons project to the glomeruli of the main olfactory bulb (MOB; Scalia and Winans, 1975; Crespo et al., 2013). The MOS responds to thousands of volatile chemosignals, which carry information regarding food, pathogens, prey, predators, and conspecifics (Firestein, 2001).

The receptors of the VNS are located in the microvilli lining the neuroepithelium of the vomeronasal organ (VNO; Salazar et al., 1997, 2003). The vomeronasal neurons project to the accessory olfactory bulb (AOB; McCotter, 1912; Halpern et al., 1995). Among mammalian groups expressing the two vomeronasal receptors families, V1R and V2R (Herrada and Dulac, 1997), a morphofunctional antero-posterior subdivision is established within the AOB (Shinohara et al., 1992; Torres et al., 2022). The sensory neurons of the VNO detect a range of non-volatile natural ligands found in the exocrine secretions of conspecifics, which are involved in innate socio-sexual behaviors (Krieger et al., 1999; Villafranca-Faus et al., 2021).

In addition to the MOS and VNS, a range of sensory olfactory subsystems, including the Grüneberg ganglion (GG), the septal organ of Masera (SO), and guanylyl cyclase-D-expressing (GC D+) chemosensory neurons, have been characterized, primarily in laboratory rodents (Zimmerman and Munger, 2021). The GG (Grüneberg, 1973) consists of cells located in the dorsal region of the nasal vestibule that are immunopositive for olfactory-marker-protein (OMP) and are involved in the detection of highly membrane-permeant stimuli (Breer et al., 2006). GG cells project to the dorsal regions of the caudal MOB near the AOB (Fuss et al., 2005; Storan and Key, 2006). The SO is an isolated patch of sensory epithelium identified in Rodentia and located near the base of the nasal septum at the entrances to the nasopalatine ducts (Adams, 1972). The SO expresses 50–80 genes in the OR family (Kaluza et al., 2004). These neurons project to a small cluster of glomeruli located in the caudal, ventromedial aspect of the MOB (Levai et al., 2003).

The GC-D+ chemosensory neurons are a subpopulation of olfactory neurons that project to the well-defined necklace glomeruli, the most caudal glomeruli in the MOB. The necklace glomeruli, identified by Shinoda et al. (1993), were first defined in rats as a subset of OSNs immunoreactive to human placental antigen X-P2 (PAX) that converge on 7–9 glomeruli. These glomeruli overlap with a subset of “atypical” glomeruli with acetylcholinesterase (AChE) reactivity (Zheng et al., 1987; Weruaga et al., 2001). The projections from the GG and SO also overlap with the necklace complex. Although this receptor system remains minimally studied and poorly understood (Zimmerman and Munger, 2021), GC-D+ chemosensory neurons are not OMP+ and function as receptors for uroguanylin, guanylin, and urine components (Leinders-Zufall et al., 2007).

All known olfactory subsystems converge onto the transition zone located between the MOB and the AOB. This transition zone is a modified bulbar cortex, bounded anteriorly by the dorsal MOB and posteriorly by the anterior AOB (Larriva-Sahd, 2012) and has been named the olfactory limbus (OL). The OL contains specialized glomeruli that receive noncanonical sensory afferents and interact with the MOB and AOB, indicating that the OL may serve as an integration site for non-olfactory and atypical vomeronasal sensory inputs (Vargas-Barroso et al., 2017).

Little is known regarding the olfactory subsystems present in carnivores. No sensory systems equivalent to the SO, GG, or necklace glomeruli have been described in either dogs or cats (Salazar and Sánchez-Quinteiro, 2011; Barrios et al., 2014b). However, studies characterizing the AOB in minks, meerkats, and dogs have indicated a more complex glomerular organization in the OB than has been described for other mammalian orders. The presumptive AOB in minks comprises not only the main dorsocaudal protuberance but also smaller lateral and medial regions (Salazar et al., 1998), whereas a recent study of the meerkat OB found a subpopulation of atypical glomeruli with a strong calretinin (CR)-positive neuropil in the vicinity of the AOB (Torres et al., 2021). A study by Miodonski in the dog (1968) described the AOB as consisting of distinct glomerular aggregations, both “in the dorsomedial side of the OB, at the posterior margin of the MOB and downward along the caudal edge of the MOB, descending to its base both on the medial and on the lateral side.” Miodonski’s observations may represent the erroneous attribution of atypical glomerular structures to the accessory olfactory system instead of alternative olfactory subsystems; however, the multiform characterization of the dog AOB was not confirmed by subsequent lectin and immunohistochemical studies (Nakajima et al., 1998; Salazar et al., 2013). Only Nakajima et al. (1998) identified a small group of glomeruli with NADPH-diaphorase reactivity “between the glomeruli at the most caudal portion of the MOB, ” which they attributed to the specific projections from subsets of neurochemically distinct OR cells.

Recent studies of the AOB in wild canids, including the African wild dog (*Lycaon pictus*; Chengetanai et al., 2020) and the red fox (*Vulpes vulpes*; Ortiz-Leal et al., 2022a) indicate significant differences in the AOB between domestic and wild canids. In the red fox, a small atypical glomerular formation was identified in the proximity of the AOB. In the present study, we focused on characterizing the OL in the red fox by performing general and specific histological stainings on serial sections, using both single and double immunohistochemical and lectin-histochemical labeling techniques. We describe an OL in the red fox that displays an uncommonly high degree of development and complexity, suggesting that the red fox may represent a novel mammalian model, the study of which could improve our understanding of the noncanonical pathways involved in the processing of chemosensory cues.

## Materials and Methods

### Samples

For this investigation, three male and two female adult red foxes (*Vulpes vulpes*) were employed. They were obtained through hunting expeditions led by the Galician Hunting Federation with the required authorizations granted by the Galician Environment, Territory, and Tenement Council. The animals were brought to the Veterinary Faculty of Lugo’s facilities as soon as they were shot, in the field, with no more than a 2-h interval. There, the rostral section of the encephalon was removed using an electric plaster cutter and a gouge clamp and preserved in Bouin’s fixative (Bf). Afterwards, the olfactory bulbs in conjunction with the rostral frontal lobes were embedded in paraffin wax and serially cut by a microtome in a horizontal plane along its entire length with a thickness of 6–7 μm. Hematoxylin-eosin and Nissl stains, lectin histochemistry, and both single and double immunohistochemistry were used to stain the slides.

### Lectin histochemistry staining

*Ulex europaeus* agglutinin lectin (UEA) was employed as a first step in some of the sequential double immunohistochemical labelings. UEA labels the VNS pathway in several species, including the fox (Ortiz-Leal et al., 2022a) and dog (Salazar et al., 1994).

All sample slides were deparaffinized and rehydrated prior to beginning the lectin histochemistry procedure. The samples were then incubated in 3% H_2_O_2_ solution for 15 min to inhibit endogenous peroxidase activity, followed by two rinses in pH.7.2, 0.1 M phosphate buffer (PB). Then, sections were incubated for 30 min in a 2% BSA solution, which blocked non-specific binding. The slides were then washed three times for 5 min in a PB solution, followed by an incubation period of 1 h at room temperature in a 0.5% BSA/UEA solution. Further overnight incubation with an anti-UEA peroxidase-conjugated antibody was performed on the slides. The samples were rinsed in 0.2 M Tris-HCl, pH 7.61 for 10 min the next day, followed by a PB wash, before being developed using a Diaminobenzidine (DAB) chromogen. Controls involved removing the UEA and allowing the lectin to be preabsorbed by employing an excessive quantity of the matching sugar, L-fucose.

### Simple immunohistochemical staining

The fox olfactory limbus was examined in-depth using immunohistochemistry. Among the antibodies employed (Table 1), the anti-Gαo and anti-Gαi2 antibodies are particularly useful because they label the transduction cascade for V2R and V1R vomeronasal receptors, respectively. Using antibodies against microtubule-associated protein 2 (MAP-2), neuronal dendritic development in the olfactory limbus was studied. Prior research has shown that the distribution of calcium-binding proteins may be used as a neuronal marker to distinguish between distinct brain areas and neuronal subpopulations (Baimbridge et al., 1992). Therefore, the calcium-binding proteins calbindin (CB), calretinin (CR), and secretagogin (SG), which play a role in controlling the levels of cytosolic free calcium ions in neurons, were studied using immunohistochemistry.

**Table 1:** Detailed information on the antibodies and lectins used in this study.

**Antibody**	**1st Ab species / dilution**	**1st Ab catalog number**	**Immunogen**	**Reference**	**RRID**	**2nd Ab species/dilution, catalog number**
Anti-Gαo	Rabbit 1:200	MBL-551	Bovine GTP Binding Protein Gαo subunit	Prince et al. (2009)	AB_591430	ImmPRESS VR HRP Anti-rabbit IgG Reagent MP-6401-15
Anti-Gαi2	Rabbit 1:200	Santa Cruz Biotechnology sc-7276	Peptide mapping within a highly divergent domain of Gαi2 of rat origin	de la Rosa-Prieto et al. (2010)	AB_2111472	ImmPRESS VR HRP Anti-rabbit IgG Reagent MP-6401-15
Anti–MAP-2	Mouse 1:400	Sigma M4403	Rat brain microtubule-associated proteins	Kotani et al. (2010)	AB_477193	ImmPRESS VR HRP Anti-mouse IgG Reagent MP-6402-15
Anti—CB	Rabbit 1:6,000	Swant CB38	Rat recombinant calbindin D-28k	Hermanowicz-Sobieraj et al. (2018)	AB_10000340	ImmPRESS VR HRP Anti-rabbit IgG Reagent MP-6401-15
Anti-CR	Rabbit 1:6,000	Swant 7697	Recombinant human calretinin containing a 6-his tag at the N-terminus	Adrio et al. (2011)	AB_2619710	ImmPRESS VR HRP Anti-rabbit IgG Reagent MP-6401-15
Anti-SG	Rabbit 1:1,000	Gift from Ludwig Wagner (University of Vienna, Austria)	Recombinant human secretagogin	Alpár et al. (2012)	AB_1079874	ImmPRESS VR HRP Anti-rabbit IgG Reagent MP-6401-15
UEA	1:60	Vector L-1060			AB_2336767	Rabbit 1:50 DAKO P289

Abbreviations: ABC, avidin-biotin complex; CB, calbindin; CR, calretinin; HRP, horseradish peroxidase; MAP-2, microtubule-associated protein 2; SG, secretagogin; UEA, *Ulex europaeus* agglutinin.

#### Antibody characterization and specificity

Table 1 provides details for all antibodies, including their suppliers, dilutions, target immunogens, and Research Resource Identifiers (RRID). In every instance, the immunostaining patterns produced in the red fox using these antibodies matched those previously obtained in a number of mammalian species. Table 1 lists relevant references for each antibody.

#### Simple immunohistochemical protocol

Deparaffinized and rehydrated samples were treated for 15 min in a 3% H_2_O_2_ solution to inactivate endogenous peroxidase activity prior to the immunohistochemistry reaction. To prevent non-specific binding sites, 2.5% horse normal serum from the ImmPRESS reagent kit Anti-mouse IgG/Anti-rabbit IgG (Vector Laboratories, Burlingame, CA, USA) was used for 30 min (Table 1). After that, the samples were treated with the primary antibody for an overnight incubation at 4°C with humidity. Using either the ImmPRESS VR Polymer HRP Anti-Rabbit IgG or the Anti-Mouse IgG reagent, samples were incubated for 30 min (Table 1). In every instance, 3 × 3 min PB washes were carried out in between steps. Finally, slides were developed with a DAB chromogen (using the same procedure as for the lectin histochemical labeling) and then dehydrated and mounted.

The omission of the primary antibody was employed as a negative control for all immunohistochemistry methods, and none of the negative control samples showed any labeling or non-specific background staining. We repeated the immunohistochemistry process on previously unstained mouse or rabbit tissue from earlier investigations as a positive control. These samples were all known to express the desired proteins, and each time, the expected positive results were obtained.

#### Double-immunohistochemical protocol

A sequential twice-repeated enzyme-labeled approach was used for double immunostaining (Hasui et al., 2003). The sections were given a 5-min dip in 0.1 M glycine solution (pH 2.2) in between both immunolabelings. To select the most suitable dye to visualize the immunoreaction, both DAB and Vector VIP Peroxidase Substrate Kit (SK-4600, Vector Laboratories) were combined exchanging their order. Using first DAB and then VIP was the optimal combination for our immunostaining.

### Image acquisition and digital processing

A Zeiss Axiophot microscope and a Karl Zeiss Axiocam MRc5 digital camera were used for image acquisition. Using Adobe Photoshop CS4 the white balance settings were adjusted (Adobe Systems, San Jose, CA, USA). No particular details were added, relocated, improved, or removed from the photographs.

## Results

**Figure 1:**
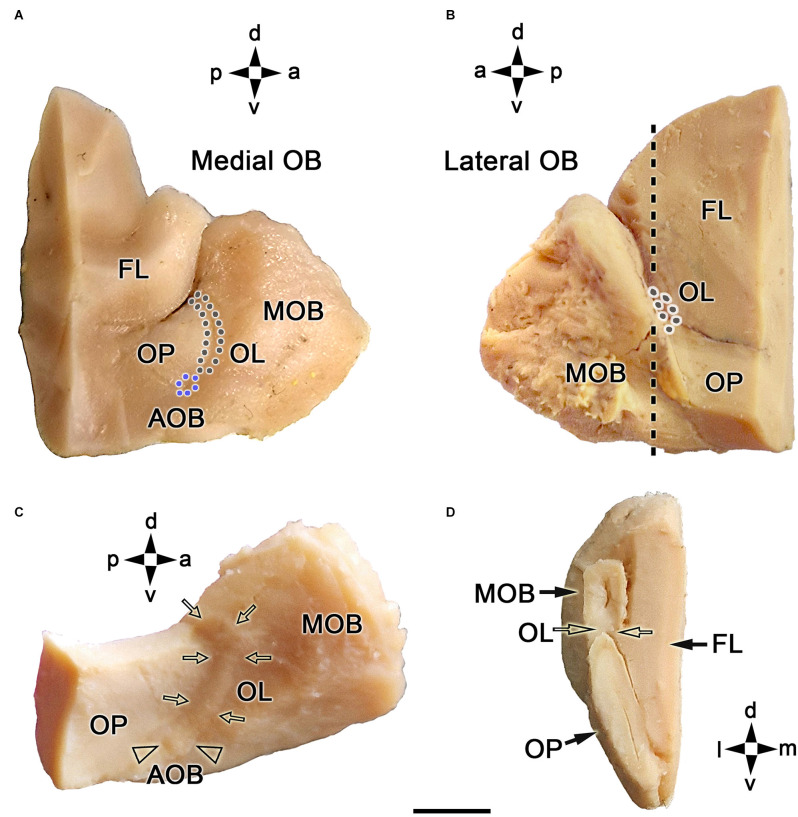
Macroscopic topographical anatomy of the fox olfactory limbus (OL). **(A)** Medial view of the left main olfactory bulb (MOB). The olfactory peduncle (OP) and the caudomedial margin of the olfactory bulb are visualized. The accessory olfactory bulb (AOB, blue dots) and the olfactory limbus (OL, black dots) are located along the MOB’s caudal surface. **(B)** Lateral view of the left main olfactory bulb. The lateral most portion of the OL is located in the caudal margin of the MOB. **(C)** Dorsolateral view of the right MOB after removal of the frontal lobe (FL). The entire length of the OL (arrows) and the AOB (arrowheads) can be visualized. **(D)** Caudal view of a transverse section of the left MOB and OP at the level depicted by a dashed line in **(B)**. The OL is located in the caudoventral portion of the MOB (open arrows). a, anterior; d, dorsal; l, lateral; m, medial; p, posterior; v, ventral. Scale bar: 500 μm.

Both macroscopic and microscopic studies were performed on the OB to describe the topographical anatomy of the fox OL (Figure 1). The OL is mainly located along the caudomedial margin of the OB, rostrally to the olfactory peduncle (Figure 1A). A minor portion projects along the caudolateral margin of the OB (Figure 1B). The entire length of the OL and AOB can be better visualized on a dorsolateral view of the OB after removing the telencephalic frontal lobe (Figure 1C). The AOB is located caudoventrally to the OL, on the OB medial surface. A transverse section of the OB at the level of the OL (Figure 1B) shows from a caudal viewpoint the topographic relationship between the OL, the MOB, and the olfactory peduncle (OP; Figure 1D).

**Figure 2:**
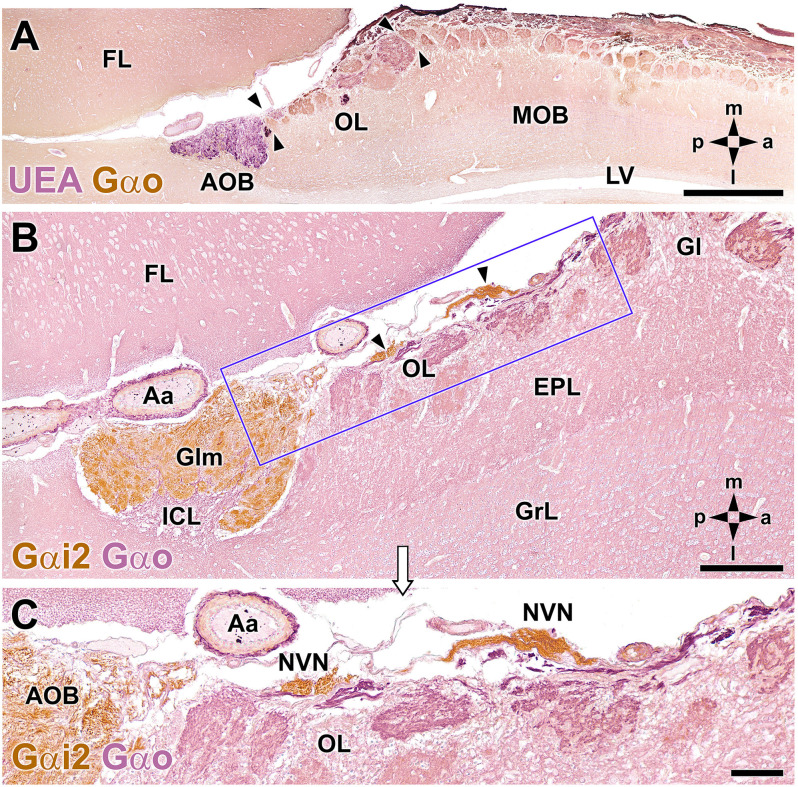
Double-immunohistochemical labeling of the fox olfactory limbus (OL). **(A)** Double immunostaining with *Ulex europaeus* agglutinin (UEA) lectin (magenta) and Gαo antibody (brown). The vomeronasal nerve and glomerular layers of the accessory olfactory bulb (AOB) are strongly labeled with UEA. Anti-Gαo immunolabeling shows a widespread immunopositive pattern, more intensely in both the olfactory nerve and glomerular layers of the MOB. The OL, delimited by arrowheads, comprises irregularly shaped glomeruli, without a homogeneous immunostaining pattern. **(B,C)** Double immunostaining for Gαi2 (brown) and Gαo (magenta). Anti-Gαi2 stains the superficial AOB and the *nervus vomeronasalis* (NVN, arrowheads). Anti-Gαo stains the internal cellular layer (ICL) of the AOB and the neuropil of the olfactory bulb. The box in **(B)** is enlarged in **(C)**, showing that the OL comprises irregularly shaped glomeruli without a homogeneous immunostaining pattern. The branches of the NVN, which are Gαi2-immunopositive, contrast with the Gαo-immunopositive nerve endings that project to the OL glomeruli. Aa, artery; EPL, external plexiform layer; FL, frontal lobe; Gl, MOB glomerular layer; Glm, AOB glomerular layer; GrL, MOB granular cell layer; LV, lateral ventricle. The compass indicates the orientation, as follows: m, medial; l, lateral; p, posterior; a, anterior. Scale bars: **(A)**: 1 cm. **(B,C)**: 250 μm.

A microscopic study of the fox OL was performed on serial, horizontal sections obtained from the anterior region of the telencephalon across an area that includes the AOB, MOB, and frontal lobe (FL). The OL was defined as the transition zone between the MOB and AOB, consisting of a large area located in the medial OB, delimited by the dorsocaudal edge of the MOB and the anterior end of the AOB (Figure 2). The OL is not a sharp frontier but is instead a heterotypical bulbar cortex with distinct cytological characteristics relative to the MOB and AOB. A laminar pattern can be observed throughout the OB, whereas the OL features varying degrees of lamination, which is its most obvious characteristic. The widespread presence of atypical glomeruli in the fox OL is remarkable (Figures 2B,C). In contrast to the linear arrangement and uniform size of glomeruli in the MOB (100–150 μm maximum diameter), glomeruli in the OL are characterized by an atypical, disarrayed arrangement, irregular shapes, and a wide range of sizes (50–500 μm maximum diameter).

Double immunohistochemical labeling against different markers allowed us to characterize the topographical relationships between the atypical glomeruli in the OL (Figure 2A), the AOB, and the axonal endings of the vomeronasal nerve (NVN; Figure 2B). Double immunolabeling against *Ulex europaeus* agglutinin (UEA) lectin and the G protein subunit Gαo (Figure 2A) identified two superficial layers in the AOB, the NVN, and glomerular layers, both of which were intensely stained by UEA. Conversely, anti-Gαo staining was observed in the deep layers of the AOB and throughout the MOB, with more intense staining observed in both the olfactory nerve and glomerular layers. Strikingly, the OL, delimited by arrowheads in Figure 2A, was comprised of irregularly shaped glomeruli with no homogeneous immunostaining pattern.

Double immunolabeling against the G protein subunits, Gαo and Gαi2 (Figure 2B), is equally useful to double staining with UEA and Gαo. Gαi2 is expressed exclusively in the VNS, at both the AOB surface and in the NVN (Figure 2C). By contrast, the nerve endings projecting to the OL glomeruli were Gαo-immunopositive (Figure 2C).

**Figure 3:**
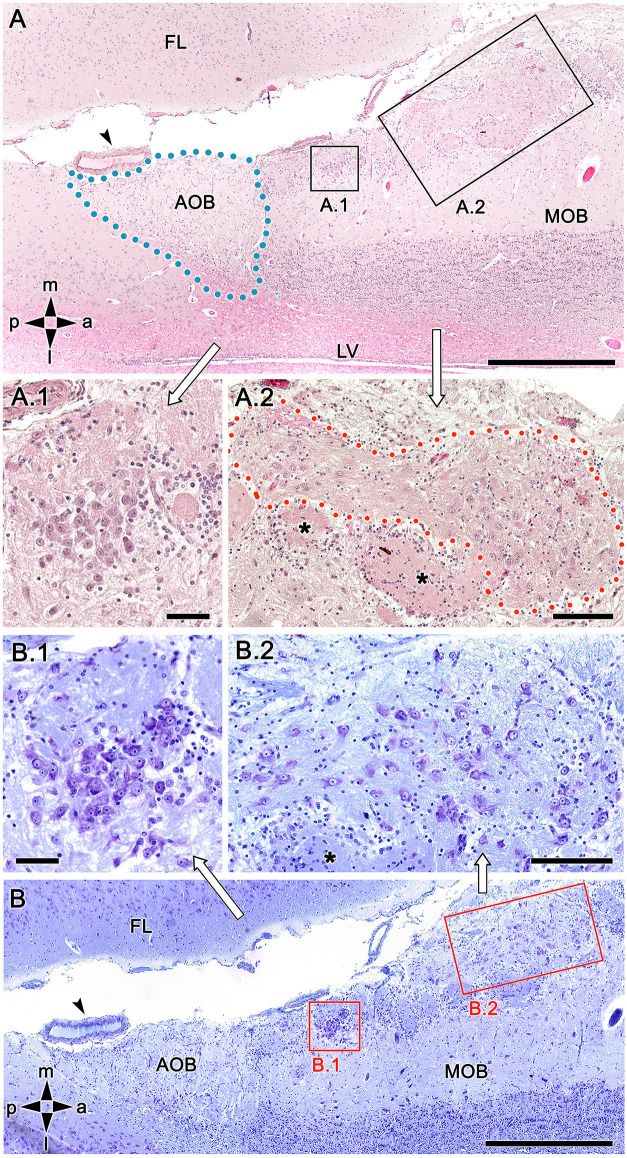
Histological study of the fox olfactory limbus (OL). **(A)** General view of the OL, in the horizontal plane. The accessory olfactory bulb (AOB), close to a large artery (arrowhead), is framed by dots. The two most strikingly discernible features of the OL are framed and shown at higher magnification in **(A.1)** and **(A.2)**: the dense neuronal cluster **(A.1)** and the macroglomerular complex (MGC, delimited by red dots in **A.2**), a broad nervous formation consisting of neuronal somata distributed within a neuropil with clearly distinct boundaries. Small, irregularly shaped glomeruli were observed deep to both structures (asterisks). **(B)** Consecutive, Nissl-stained serial sections showing the morphology of the neuronal somata. Both the denser aggregates **(B.1)** and the MGC **(B.2)**, possess polygonal, ellipsoidal, and rounded somata. FL, frontal lobe; LV, lateral ventricle; MOB, main olfactory bulb. Orientation: m, medial; l, lateral; p, posterior; a, anterior. Scale bars: **(A,B)**: 500 μm. **(A.1,B.1)**: 100 μm. **(A.2,B.2)**: 250 μm.

Using both hematoxylin-eosin (Figure 3A) and Nissl staining (Figure 3B) revealed the OL texture, which is characterized by high structural complexity. The two most striking features are dense, compact clusters of neuronal somata (Figures 3A.1,B.1) and a broad nervous formation consisting of less densely aggregated neuronal somas distributed within a neuropil that is distinct from the underlying glomeruli (Figures 3A.2,B.2). We have termed this previously undescribed nervous formation the macroglomerular complex (MGC). Both features are characterized by a remarkably high concentration of neuronal somata located in the most superficial bulb layer, proximal to the pial surface. Small, irregularly shaped glomeruli deep into both structures complete the organization of the OL.

**Figure 4:**
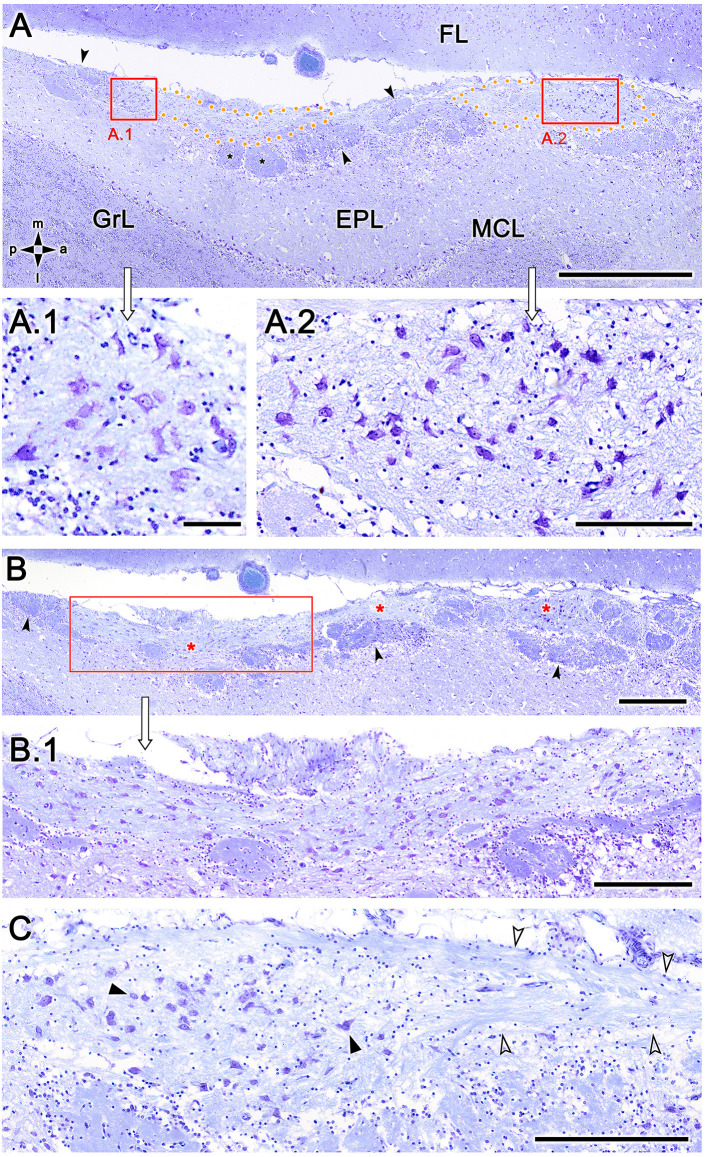
Nissl-stained sections of the fox olfactory limbus (OL) at different horizontal levels. Serial Nissl-stained sections from a single specimen reveal the development of the OL in this species. **(A)** A neuronal cluster (box enlarged in **A.1**) and the superficial macroglomerular complex (MGC, encircled by yellow dots and partially enlarged in **A.2**) were observed in a section at the ventral level. Most glomeruli proximal to the MGC had irregular and atypical shapes (arrowheads), but some appeared spherical (black asterisk). **(B)** A more dorsal section shows the extent of the MGC (red asterisks). The red box is shown at a higher magnification in **(B.1)**. Atypical glomeruli were also observed (arrowheads). **(C)** An even more dorsal section shows the morphologies of MGC neurons (black arrowheads) and their association with a prominent fascicle of nerve fibers (open arrowheads). FL, frontal lobe of the telencephalon; GrL, granular layer; EPL, external plexiform layer; MCL, mitral cell layer. Orientation: m, medial; l, lateral; p, posterior; a, anterior. Scale bars: **(A)**: 1 mm. **(B)**: 500 μm. **(A.2,B.1,C)**: 250 μm. **(A.1)**: 100 μm.

**Figure 5:**
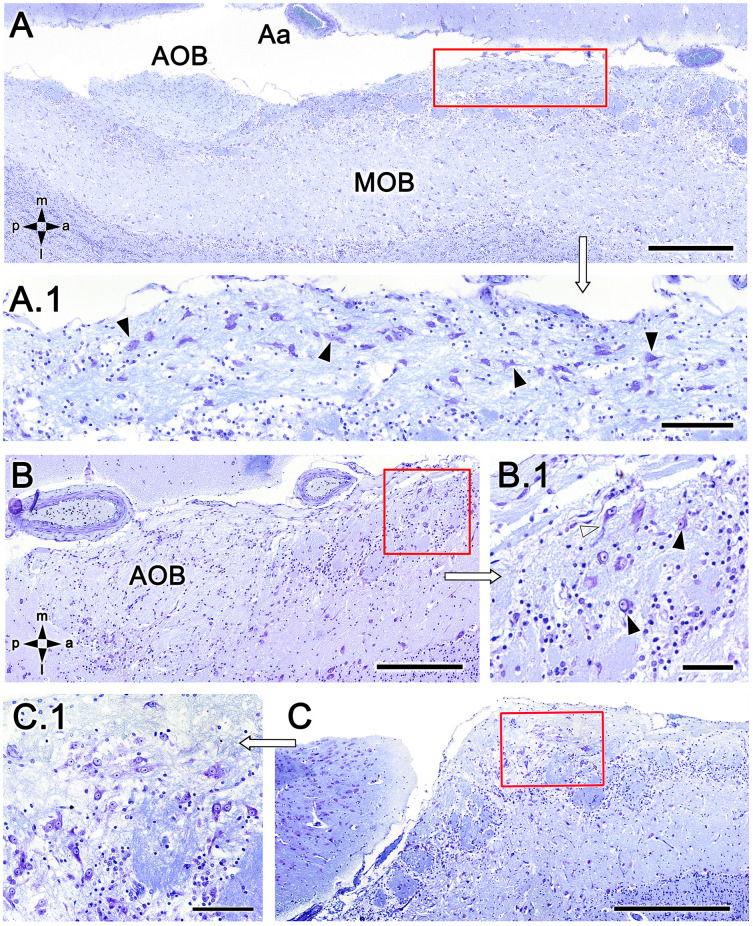
Nissl-stained sections of the fox olfactory limbus (OL). **(A)** A ventral section shows the topography of the nervous formation, located on the bulbar surface, superficial to the glomeruli. **(A.1)** A higher magnification of the red box in **(A)** shows numerous neuronal somata (arrowheads). **(B)** Horizontal section at the level of the AOB. Anterior to the AOB, a superficial neuronal cluster surrounded by atypical glomeruli can be observed (red box, magnified in **B.1**). **(B.1)** The neuronal somata have oval shapes (arrowheads), and the origin of thick processes is visible (open arrowhead). **(C)** In this specimen, the neuronal cluster is located in a more anterior position, very close to the pial surface. **(C.1)** At a higher magnification, the neurons show similar morphology to that of the specimen shown in **(B)**. Aa, artery; AOB, accessory olfactory bulb; MOB, main olfactory bulb. Orientation: m, medial; l, lateral; p, posterior; a, anterior. Scale bars: **(A,C)**: 500 μm. **(B)**: 250 μm. **(A.1,C.1)**: 100 μm. **(B.1)**: 50 μm.

To exclude the possibility that these atypical formations are the result of interindividual anatomical variability, we performed Nissl staining in four additional animals, as shown in Figures 4 and 5. In the first specimen (Figure 4), horizontal OL sections were stained at three levels dorsal to the AOB, which allowed for the detailed characterization of the appearance and dimensions of both neuronal clusters (Figures 4A,A.1) and the MGC (Figures 4A,A.2,B,B.1,C). Somata in the clusters were similarly sized, with polyhedral, ellipsoid, or oval morphologies (Figures 4A.1,A.2,B.1,C). The MGC was always located on the bulbar surface, close to glomeruli populations located in the immediately deeper plane, and the magnitude of the MGC was notable, extending over several millimeters in some sections (Figures 4A,B). Most of the glomeruli associated with the MGC featured irregular, atypical shapes (Figures 4A,B), although some glomeruli with typical spherical shapes and well-defined boundaries were also observed (Figure 4A). Nissl staining of the other three individuals (Figures 5A–C) verified these findings.

**Figure 6:**
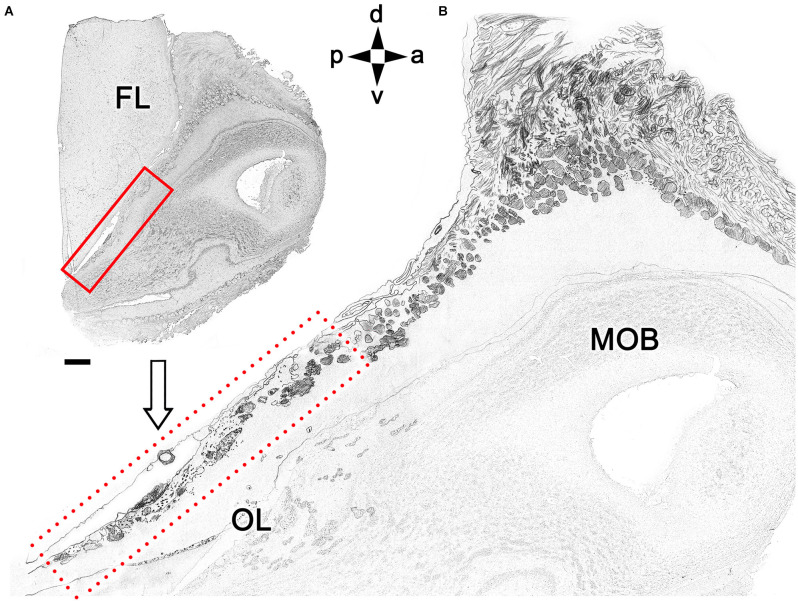
Schematic drawing of a sagittal histological section of the olfactory bulb. **(A)** Magnification of the area corresponding to the OL (box in **B**). Orientation: m, medial; l, lateral; p, posterior; a, anterior. Scale bar: 500 μm.

Figure 6 summarizes our interpretation of the histological data in a schematic drawing of a histological MOB section (Figure 6A) and an enlargement of the caudodorsal area, showing the irregular arrangement and dimensions of atypical glomeruli and the structure of the MGC (Figure 6B). To further characterize the fox OL neurochemically and identify potentially distinctive features relative to the AOB and MOB, we conducted an immunostaining study using various antibodies and the lectin UEA.

**Figure 7:**
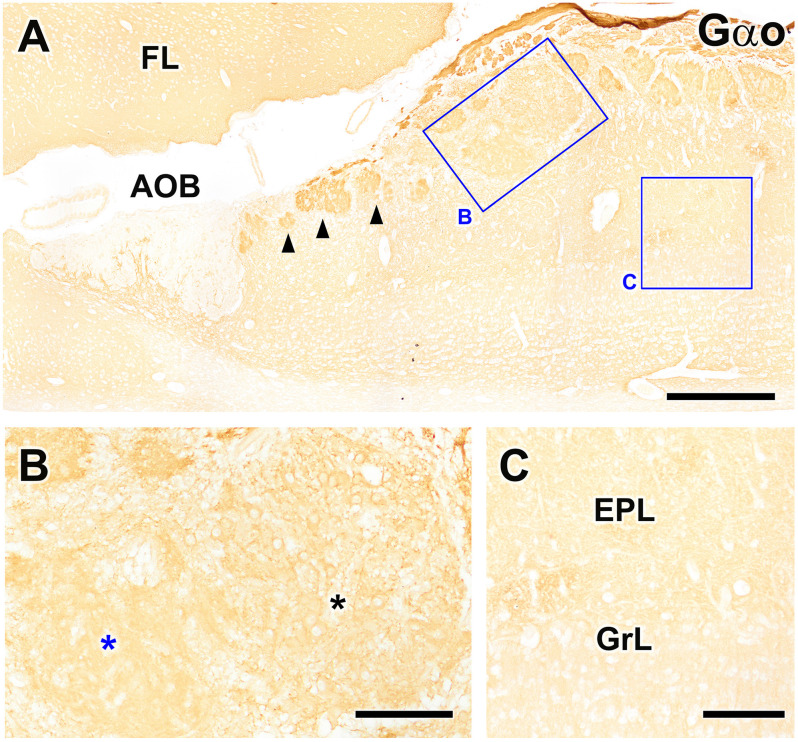
Immunohistochemical labeling of the fox olfactory limbus (OL) using anti-Gαo. **(A)** Immunolabeling for the G-protein subunit Gαo revealed uniform expression of Gαo throughout the frontal lobe of the telencephalon (FL) and the olfactory bulb, but no immunostaining in the nerve or glomerular layers of the accessory olfactory bulb (AOB). Atypical glomeruli proximal to AOB (arrowheads) are more intensely immunolabeled with anti-Gαo than typical glomeruli in the main olfactory bulb (MOB). The macroglomerular complex (MGC, box **B**) shows a labeling intensity similar to the typical MOB glomeruli. **(B)** Higher magnification of box **(B)**, showing that the MGC possesses two clearly differentiated areas: one containing numerous neuronal somata (black asterisk) and one devoid of somata (blue asterisk). Somata in the MGC are Gαo-immunopositive, unlike the mitral cell somata in the MOB, which are immunonegative (box **C**, **C**). EPL, external plexiform layer; GrL, granular layer. Scale bars: **(A)**: 500 μm. **(B,C)**: 125 μm.

Immunolabeling against the G protein subunit Gαo resulted in the well-characterized pattern of immunopositivity throughout the neuropil of the FL and OB, with Gαo immunonegativity observed only in the nerve and glomerular layers of the AOB (Figure 7A). However, the atypical glomeruli proximal to the AOB showed more intense immunolabeling than the typical glomeruli of the MOB. The MGC showed a labeling intensity similar to that observed for typical glomeruli. When the MGC was observed at higher magnification (Figure 7B), two clearly differentiated areas could be identified, one containing numerous Gαo-immunopositive neuronal somata and the other devoid of them, in contrast with the Gαo-immunonegative mitral cell somata of the MOB (Figure 7C).

**Figure 8:**
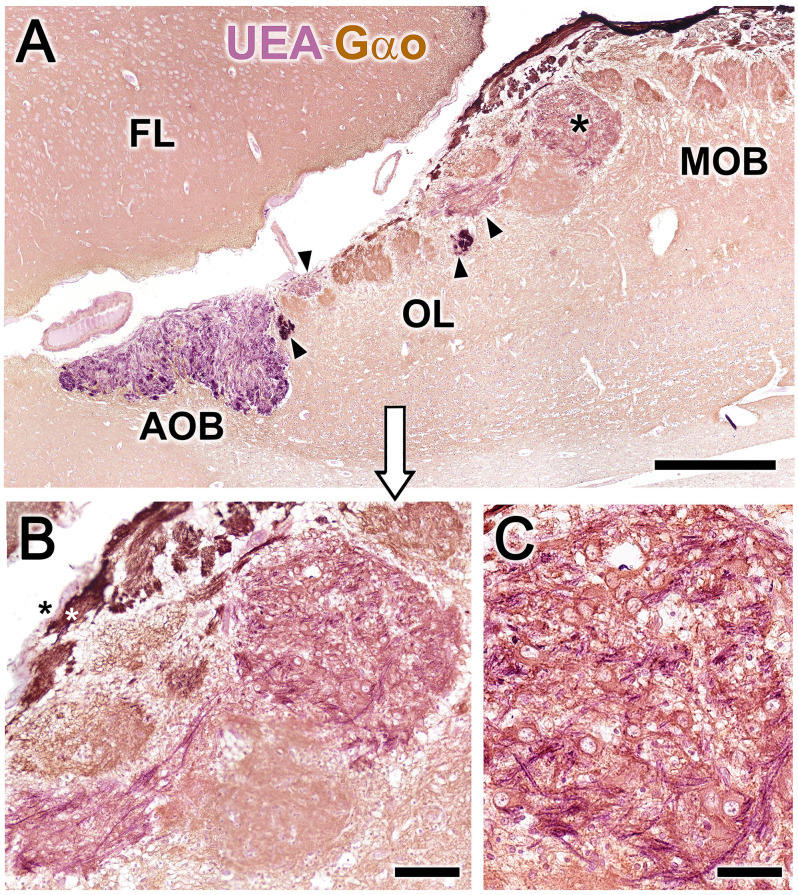
Double-immunohistochemical labeling of the fox olfactory limbus (OL). Double immunostaining with the lectin *Ulex europaeus* agglutinin (UEA, magenta) and Gαo antibody (brown). **(A)** The nerve and glomerular layers of the accessory olfactory bulb (AOB) are intensely labeled by UEA, whereas the main olfactory bulb (MOB) is not. In the OL, some glomeruli are UEA-negative, whereas smaller glomeruli are strongly UEA-positive (arrowheads). The macroglomerular complex (MGC, asterisk) is also UEA-positive. **(B)** Higher magnification of the MGC area, showing Gαo-positive somata embedded in a dense network of UEA-positive fibers. The vomeronasal nerve fibers segregate into UEA-positive (black asterisk) and Gαo-positive (white asterisk components). **(C)** The MGC somata are oval in shape and Gαo-positive. FL, Frontal lobe of the telencephalon. Scale bars: **(A)**: 500 μm. **(B)**: 50 μm.

We returned to the original double immunolabeled sections shown in Figure 2 to more closely examine the structural organization of the MGC. Figure 8 shows higher magnifications of the double immunolabeled OL sections presented in Figure 2. In Figures 8A,B, the nerve and glomerular layers of the AOB appear intensely labeled by the lectin UEA, whereas the MOB glomeruli are not labeled. In the OL, some glomeruli are UEA-negative, whereas the remaining, primarily smaller glomeruli, are strongly labeled with UEA (Figure 8A). Remarkably, Gαo-positive somata belonging to the nervous formation are embedded in a dense network of UEA-positive fibers (Figures 8B,C). The NVN fibers segregate into UEA-positive (Figure 8B, black asterisk) and Gαo-positive (Figure 8B, white asterisk) components.

**Figure 9:**
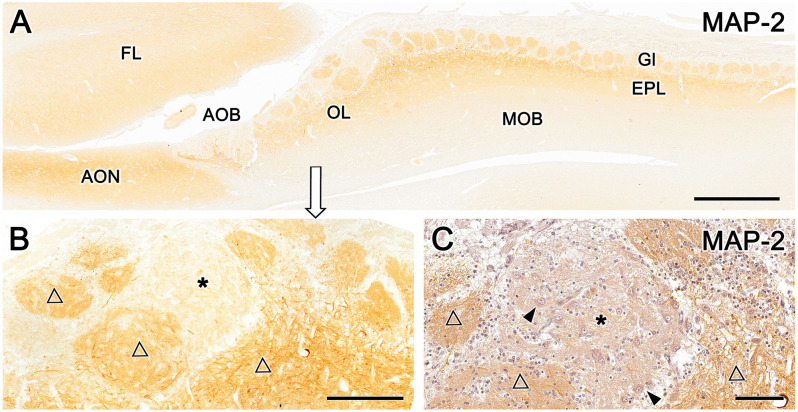
Immunohistochemical labeling of the fox olfactory limbus (OL) with anti-microtubule-associated protein 2 (MAP-2). **(A)** Immunostaining with anti-MAP-2 results in the strong and uniform immunolabeling of glomeruli (Gl) and the external plexiform layer (EPL) of the main olfactory bulb (MOB). **(B)** A higher magnification view of the rostral part of the OL shows the staining of atypical Gl formations (open triangles), in addition to a patch of the nervous formation that remains unstained (*). **(C)** Anti-MAP-2 immunostained serial sections were counterstained with hematoxylin, confirming that the unlabeled area corresponds to an area of the macroglomerular complex that is rich in neuronal bodies (arrowheads) and receives the innervation from Ulex europaeus agglutinin-positive fibers. AOB, accessory olfactory bulb; AON, anterior olfactory nucleus; EPL, external plexiform layer; FL, front lobe of the telencephalon; Gl, glomeruli; MOB, main olfactory bulb; OL, olfactory limbus. Scale bars: **(A)**: 1 mm. **(B)**: 250 μm. **(C)**: 100 μm.

Immunolabeling with the anti-microtubule-associated protein 2 (MAP-2) antibody, a consistent marker for the dendritic branching of neuronal cells in all species, results in the uniform staining of glomeruli and the external plexiform layer of the MOB (Figure 9A). In the OL, anti–MAP-2 also stains atypical glomerular formations, but a patch of the MGC remains unstained (Figure 9B, asterisk). Counterstaining of an immunolabeled section with hematoxylin (Figure 9C) confirmed that the unlabeled area corresponds to the superficial area rich in neuronal bodies receiving UEA-positive innervations, shown in Figure 8B.

**Figure 10:**
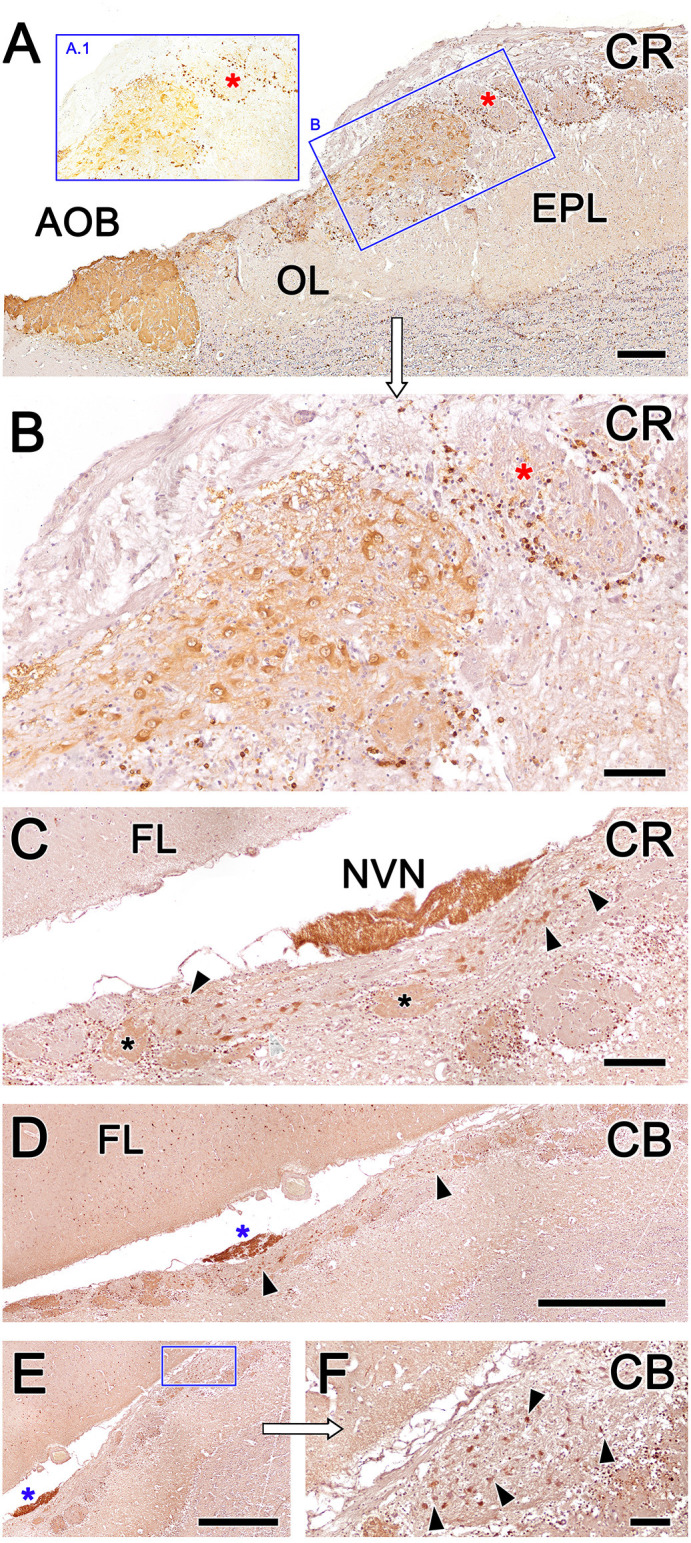
Fox olfactory limbus (OL) sections immunolabeled with the calcium-binding proteins calretinin (CR) and calbindin (CB) and counterstained with hematoxylin.** (A)** Anti-CR immunostaining produces intense labeling in the macroglomerular complex (MGC) of the OL (box **B**) and in the accessory olfactory bulb (AOB). The glomeruli of the main olfactory bulb (MOB) do not show any immunolabeling in their neuropil (red asterisks), although their periglomerular cells are clearly labeled, which is particularly evident in non-counterstained sections (box **A.1**). **(B)** A higher magnification image of box **(B)** from panel **(A)**. In the MGC, both neuronal somata and the neuropil (asterisk) are immunolabeled with anti-CR. **(C)** Section at the level of the vomeronasal nerve (NVN) shows intense anti-CR immunopositivity. The atypical nervous formation beneath the NVN is elongated and contains scattered neuronal somata (arrowheads). A subpopulation of glomeruli is CR-positive (asterisks). **(D)** Anti-CB immunostaining produces a similar pattern to anti-CR immunostaining. The NVN is intensely immunopositive to anti-CB (blue asterisk). The MGC is delimited by arrowheads. **(E)** Atypical glomeruli are variably labeled with anti-CB. **(F)** Higher magnification of the inset in **(E)**. Somata belonging to the MGC are intensely stained (arrowheads). AOB, accessory olfactory bulb; OL, olfactory limbus; EPL, external plexiform layer; FL, the frontal lobe of the telencephalon; NVN, vomeronasal nerve Scale bars: **(D,E)**: 1 mm. **(A,C)**: 250 μm. **(B,F)**: 100 μm.

**Figure 11:**
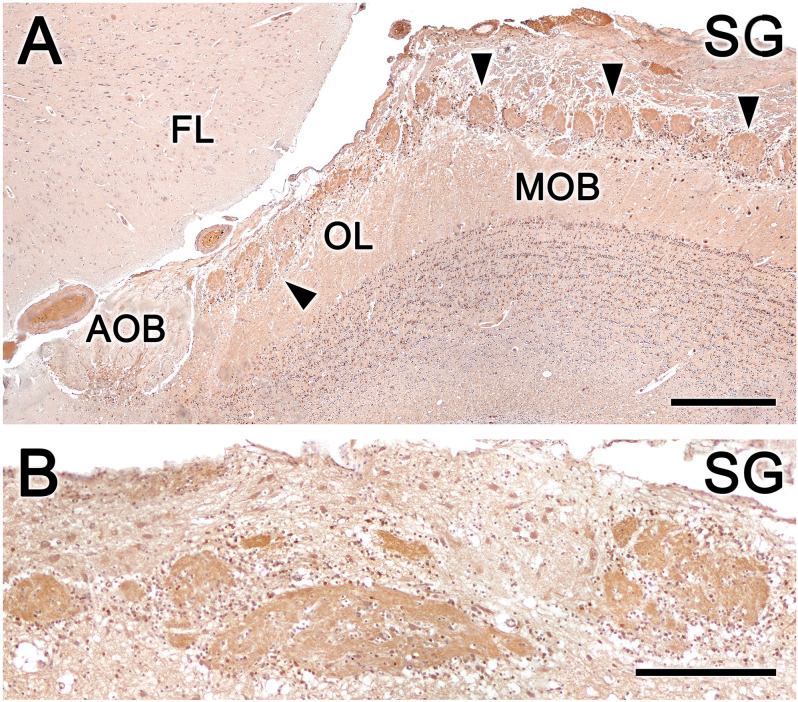
Fox olfactory limbus (OL) sections immunolabeled for the calcium-binding protein secretagogin (SG) and counterstained with hematoxylin. **(A)** Anti-SG immunostaining results in immunolabeling of the entire glomeruli population (arrowheads) in both the OL and the main olfactory bulb (MOB) but not the glomerular layer of the accessory olfactory bulb (AOB). Periglomerular cells are intensely labeled. **(B)** In the macroglomerular complex, both the neuropil and neurons are SG-immunopositive, with variable labeling intensity. FL, the frontal lobe of the telencephalon. Scale bars: **(A)**: 500 μm. **(B)**: 250 μm.

Calcium-binding proteins are commonly used as markers in the study of the OB. We selected three markers to characterize the OL: anti-calretinin (CR, Figure 10), anti-calbindin (CB, Figure 10), and anti-secretagogin (SG, Figure 11).

Anti-CR immunostaining resulted in intense labeling in the MGC of the OL and in the AOB (Figure 10A). However, the glomeruli of the MOB did not show anti-CR immunolabeling in the neuropil, although the periglomerular cells were clearly labeled (insets A.1 and B in Figure 10A). In the MGC, both the neuronal somata and the neuropil (Figure 10B) were anti-CR immunopositive. Remarkably, anti-CR did not stain the mitral somata of the MOB. In sections performed at the level of the NVN, where the MGC is elongated and the neuronal somata are dispersed, the pattern of anti-CR immunopositivity was maintained (Figure 10C), although not all atypical glomeruli were CR-positive at this level.

The immunolabeling pattern obtained with anti-CB immunostaining was similar to that observed with anti-CR immunostaining. Somata belonging to the MGC were intensely stained, and atypical glomeruli were variably labeled, differentiating CB-positive and CB-negative subpopulations of atypical glomeruli (Figures 10D–F).

Anti-SG immunostaining produces a different immunolabeling pattern from anti-CR and anti-CB immunostaining. Anti-SG immunostaining labeled the entire glomeruli population in the OL and MOB, and periglomerular cells were intensely labeled (Figure 11A). Both the neuropil and the neurons scattered in the MGC were anti-SG immunopositive, although with varying degrees of labeling (Figure 11B).

As a summary of the results obtained, Table 2 shows an overview of the main immunohistochemical and structural features of the olfactory limbus in comparison to those of the glomeruli in both the main and accessory olfactory bulb.

**Table 2:** Comparison of immunohistochemical and structural features of the olfactory limb, and the glomeruli of the main and accessory olfactory bulb.

	**UEA**	**Gαo**	**Gαi2**	**MAP2**	**CB**	**CR**	**SG**	**Principal cells size (μm)**	**Principal cells arrangement**
OL	+++^a^	+++^a^	-	+++^a^	++	++	+++	50–500	Compact clusters/MGC
MOB Glomeruli	−	+++	−	+++	+^b^	+^b^	+++	100–150	Mitral cell layer
AOB Glomeruli	+++	−	+++	−	+++	+++	−	50–100	Internal cell layer

Abbreviations: a, Heterogeneous immunopositivity; b, Immunolabeling restricted to periglomerular cells. +++, strong positive; ++, moderate positive; +, weak expression; −, negative.

## Discussion

Prior OB investigations have primarily focused on the MOS and the VNS. Although we have made substantial advancements in our understanding of other subsystems, particularly the necklace region, which consists of a ring of interconnected glomeruli encircling the caudal end of the MOB and the anterior AOB, most research has been performed in rats and mice, and the presence and organization of olfactory subsystems in other mammalian groups remain very poorly studied. However, neuroanatomical studies of the MOB and AOB comparing laboratory rodents with other mammals, such as lagomorphs (Villamayor et al., 2020), bats (Frahm and Bhatnagar, 1980), canids (Choi et al., 2010; Ortiz-Leal et al., 2022b), artiodactyls (Park et al., 2014; Kondoh et al., 2017a), and primates (Alonso et al., 1998) have identified substantial differences in their organization between orders. Therefore, undescribed integrated olfactory subsystems may exist in other species, characterized by noncanonical morphological patterns, and these should be thoroughly investigated to comprehend interspecies differences in olfactory physiology from a solid and reliable morphological basis. The present study presents neuroanatomical, immunohistochemical, and lectin-histochemical evidence supporting the presence of an OL region characterized by a complex anatomical and morphofunctional organization that can consistently be detected in the fox, a wild Canidae model species. This finding supports the need for additional research exploring the organization of potential olfactory subsystems such as the OL in other mammalian species, including humans, as these have been poorly defined in most mammals thus far.

### The histology of the olfactory limbus of the fox

The serial histological study of fox OL revealed the presence of a complex glomerular organization extending along the area between the caudal end of the MOB and the anterior extremity of the AOB that was detected in all examined animals. To our knowledge, the studies performed by Miodonski (1968) and Nakajima et al. (1998) characterizing the dog OB represent the only descriptions of a similar atypical organization in canids. Both authors describe an unusual organization in the transition zone between the AOB and the MOB that did not extend beyond a population of atypical glomeruli, which Miodonski ascribed to the AOB and which Nakajima ascribed to the processing of information from a subset of ORs by the MOB. However, in our serial study of the fox, we identified a complex organization that goes beyond the mere presence of atypical glomeruli, which differed from typical glomeruli both in size and in neurochemical and lectin-histochemical properties. The presence of neuronal aggregates comprising large somata and nascent prolongations that were clearly visible with Nissl staining was remarkable. These somas were organized as both compact aggregates with a high neuronal density (Figures 3A.1,B.1, 4A.1) and scattered along the MGC, a superficial nervous formation extending over several millimeters consisting of a broad neuropil formed by bundles of nerve fibers (Figures 3A.2,B.2, 4A–C, 5A). The superficial location of the MGC and the presence of multiple neuronal somata inside it appear to preclude the existence of a direct relationship with the lateral olfactory tract. The presence of superficial neuronal clusters in the OB is surprising and has not been described previously, to our knowledge. Existing descriptions of atypical glomerular formations (Giannetti and Le Jeune, 1996; Gómez et al., 2005) in the necklace complex (Ring et al., 1997; Luo, 2008) and OL in rodents (Vargas-Barroso et al., 2017) do not include the presence of superficial clusters of neuronal somata similar to those detected here.

The sub-bulbar formations described by Larriva-Sahd (2012) in the rat associated with the anterior portion of the anterior olfactory nucleus and Villamayor et al. (2020) in the rabbit, associated with the AOB may be similar to the superficial clusters identified in the present study. However, these formations consist of clusters of large and polygonal neurons, differentiated by their locations in the deepest part of the caudal OB, directly associated with the anterior olfactory nucleus and the lateral olfactory tract, into which they incorporate their axons.

### Neurochemistry of the olfactory limbus of the fox

The immunohistochemical and lectin analysis performed in the present study provided an in-depth study of the neurochemical characteristics of these neurons and their associated neuropil. Both the somas and the neuropil of the atypical glomeruli were immunopositive for the Gαo G protein subunit, which is widely expressed in the OB but is absent from the mitral cell somata of the fox MOB. This feature differentiates the two systems and excludes the possibility that these cells represent ectopic MOB clusters. The extensive UEA-positive innervation observed in the MGC by double immunohistochemistry is also significant (Figure 7B). UEA is an excellent histochemical marker of α-fucose (Kondoh et al., 2017b), which serves as a VNS pathway marker in several mammalian species (e.g., dogs and pigs; Salazar et al., 1994, 2000) and mediates processes that include learning and memory, neurite outgrowth, and synaptic plasticity (Matthies et al., 1996; Kalovidouris et al., 2005; Murrey et al., 2009). We recently verified that α-fucose could be used as a VNS pathway marker in our study of the fox AOB (Ortiz-Leal et al., 2022a), and our present study of the OL reveals that UEA-positive nerve endings projecting from the NVN innervate the AOB, a subpopulation of atypical glomeruli in the OL, and Gαo-positive neuronal clusters of the MGC (Figure 7B).

MAP-2 is a useful marker for the dendritic trees of mitral and principal cells (Dehmelt and Halpain, 2005) and is not expressed in axons (Bernhardt and Matus, 1984). Anti–MAP-2 staining in the OL is, therefore, a good tool for characterizing glomeruli shapes. We found that atypical glomeruli stain in the OL more intensely for anti–MAP-2 than glomeruli in the MOB and show greater morphological diversity (Figure 7A). A similar pattern was described in a morphometric study of the mouse glomerular necklace complex (Walz et al., 2007). Another difference between the OL and the MOB is the more intense MAP-2 staining observed in MGC somata compared with mitral cell somata in the MOB (Figure 7B).

Calcium-binding proteins are widely used as markers in the study of the OB (Crespo et al., 1997; Defteralı et al., 2021), and staining patterns revealed neurochemical differences between the OL and MOB. Atypical glomeruli and neuronal aggregates in the OL were intensely stained with both CR and CB, which were observed in both the neuronal somata and neuropil. However, little labeling was observed in the neuropil of MOB glomeruli, although periglomerular cells were immunopositive. Abundant CB and CR immunopositive periglomerular cells proximal to atypical glomeruli are similar to the descriptions of the rat OB (Crespo et al., 1997). Strikingly, the principal neurons of the OL were both CB and CR immunopositive, unlike the mitral cells of the fox MOB. The absence of immunoreactivity to CR in the MOB mitral cells seems to be specific to canids as it has been described in dogs (Choi et al., 2010), wolves, and fox (Ortiz-Leal et al., 2022b), but not in other species such as rats (Wouterlood and Härtig, 1995) or meerkats (Torres et al., 2021). SG is a more recently discovered calcium-binding protein (Wagner et al., 2000) that has been less studied in the olfactory bulb than CR and CB; however, SG has been shown to be widely expressed throughout the OB layers (Kosaka and Kosaka, 2013; Pérez-Revuelta et al., 2020). In the fox, this broad pattern of SG immunolabeling was conserved in the OL and MOB but not in the AOB.

### Functional correlates for the fox olfactory limbus

The innervation of OL neuronal clusters by UEA-positive fibers establishes a clear morphofunctional link between the OL and VNS, as UEA is a highly specific marker for the fox VNS, labeling the VNS neuroepithelium, NVN, and nerve and glomerular layers of the AOB. Neuronal clusters of the OL were also Gαo-positive in both somata and the neuropil, which functionally differentiates OL neuronal clusters from the primary neurons in the MOB. in addition, different neurochemical patterns between the primary cells of the OL and the mitral cells of the MOB were identified by immunostaining for MAP-2, CB, and CR.

These morphological and neurochemical observations suggest a potential functional link between the OL and the extensive Gαo immunoreactivity observed in the fox VNO neuroepithelium (Ortiz-Leal et al., 2020). Subsequent neurochemical characterization of the fox AOB (Ortiz-Leal et al., 2022a) failed to detect Gαo immunoreactivity in the AOB nerve layer, suggesting that sensory information detected by the vomeronasal receptors associated with Gαo-positive neurons project to a different bulbar territory. The functional linkage of the OL through UEA-positive fibers projecting from the VNO, the high level of OL-associated Gαo immunoreactivity, and the topographical detection of Gαo-positive axons in the OL and the NVN (Figure 8B) lead us to hypothesize that the OL is at least partially involved in the processing of sensory information from Gαo-positive neuroepithelial cells in the VNO.

The link between the fox OL with the VNS and the strategic location of the OL between the AOB and the MOB suggest that the fox OL may be involved in the processing of specific stimuli signaling relevant intraspecific socio-sexual cues, similar to the suggested functionality of this region in laboratory rodents (Weruaga et al., 2001; Leinders-Zufall et al., 2007; Larriva-Sahd, 2012; Vargas-Barroso et al., 2017).

Our observations do not enable us to determine if the OL of the fox contains glomerular structures functionally similar to those found in rodents OL: the necklace glomeruli and the subset of “atypical” glomeruli with acetylcholinesterase (AChE) reactivity. This is a question that is still far from being answered, since the presence of the olfactory subsystems identified in rodents that project to both structures, including the Grüneberg ganglion, septal organ, and GC-D+ chemosensory neurons, has not been identified in the fox or in any other canid. The study of the expression in the fox OL of human placental antigen X-P2 (PAX), a marker of rodents’ necklace glomeruli, and acetylcholinesterase, characteristic of atypical glomeruli, could shed light on this question.

Nevertheless, features discussed in the preceding sections, such as the large development of the fox OL, both in terms of dimensions and structural complexity, and the remarkably high concentration of neuronal somata located in the most superficial bulb layer, proximal to the pial surface, suggest that the fox OL is a structure of greater complexity and functional significance than the necklace complex or the subset of atypical glomeruli described in rodents.

### Canids domestication and olfactory limbus morphology

The high degree of development and structural and neurochemical complexity found in the fox OL has not been described in the dog, which is the best-studied canid, with only the studies by Miodonski (1968) and Nakajima et al. (1998) indicating the presence of atypical glomerular organizations in the dog. However, neither of these studies described a level of structural or neurochemical complexity in the dog comparable to that observed in the fox.

The fox represents a particularly good model for researching the impacts of domestication in Canidae due to long-term experiments aimed at replicating the early domestication of this species (Belyaev et al., 1985; Wang et al., 2018). Selection for tameness resulted in similar changes in behavior, physiology, and genetic diversity in foxes as those observed in domesticated dogs (Trut et al., 2009; Kukekova et al., 2018).

Our previous studies of the fox VNS indicated the presence of striking structural differences between the dog and fox AOB, which we hypothesize might partially be the result of the domestication process (Ortiz-Leal et al., 2020, 2022a). The current theory is that the domestication of dogs has resulted in an involution of olfactory development associated with the detection of pheromones and other semiochemicals (Jezierski et al., 2016) which is supported by the anatomical differences in the fox OL and currently available descriptions of the dog OB.

## Data Availability Statement

The original contributions presented in the study are included in the article, further inquiries can be directed to the corresponding author.

## Ethics Statement

Ethical review and approval was not required for the animal study because all the animals employed in this study died by natural causes. Accordingly, the Bioethics Committee of the University of Santiago de Compostela has determined that ethics approval was not required for this study. Their report has been added to the submission.

## Author Contributions

PS-Q, AL-B, and LF collected the tissues. IO-L, MT, and PS-Q processed the tissue. IO-L, MT, PS-Q, AL-B, LF, VV-B, and JL-S analyzed and discussed the results. IO-L, MT, PS-Q, VV-B, and JL-S wrote the manuscript. All authors contributed to the article and approved the submitted version.
